# Rapid Gardos Hereditary Xerocytosis Diagnosis in 8 Families Using Reticulocyte Indices

**DOI:** 10.3389/fphys.2020.602109

**Published:** 2021-01-14

**Authors:** Véronique Picard, Corinne Guitton, Lamisse Mansour-Hendili, Bernard Jondeau, Laurence Bendélac, Maha Denguir, Julien Demagny, Valérie Proulle, Frédéric Galactéros, Loic Garçon

**Affiliations:** ^1^Service d’Hématologie Biologique, Assistance Publique-Hôpitaux de Paris, Hôpital Bicêtre, Hôpitaux Universitaires Paris-Saclay, Le Kremlin-Bicêtre, France; ^2^Faculté de Pharmacie, Université Paris-Saclay, Châtenay-Malabry, France; ^3^Service de Pédiatrie Générale, Assistance Publique-Hôpitaux de Paris, Hôpital Bicêtre, Filière MCGRE, Hôpitaux Universitaires Paris-Saclay, Le Kremlin-Bicêtre, France; ^4^Department of Molecular Genetics, Assistance Publique-Hôpitaux de Paris, Henri Mondor University Hospital, Créteil, France; ^5^Service de Biochimie, Assistance Publique-Hôpitaux de Paris, Bicêtre Hospital, Le Kremlin-Bicêtre, France; ^6^Service d’Hématologie Biologique, CHU Amiens, EA 4666 HEMATIM-UPJV, Amiens, France; ^7^Centre de Référence des Syndromes Drépanocytaires Majeurs, Hôpital Henri-Mondor, AP-HP, Créteil, France

**Keywords:** reticulocytes, Piezo1, xerocytosis, Gardos, red cell indices

## Abstract

Gardos channelopathy (Gardos-HX) or type 2 stomatocytosis/xerocytosis is a hereditary hemolytic anemia due to mutations in the *KCNN4* gene. It is rarer than inherited type 1 xerocytosis due to *PIEZO1* mutations (Piezo1-HX) and its diagnosis is difficult given the absence of a specific clinical or biological phenotype. We report here that this diagnosis can be sped up using red blood cell (RBC) indices performed on an ADVIA 2120 (Siemens^®^) analyzer, which measures reticulocyte mean corpuscular volume (rMCV) and mean corpuscular hemoglobin concentration (rMCHC). We studied reticulocyte indices in 3 new and 12 described patients (8 families) with Gardos-HX, 11 subjects presented the recurrent p.Arg352His mutation, 4 cases (two families) carried a private *KCNN4* mutation. They were compared to 79 described patients (49 families) with Piezo1-HX. Surprisingly, in Gardos-HX cases, rMCV revealed to be smaller than MCV and rMCHC higher than MCHC, in contrast with normal or Piezo1-HX RBC. Consequently, ΔMCV (rMCV-MCV) was −0.9 ± 5 fL vs. 19.8 ± 3 fL (*p* < 0.001) in Gardos compared with Piezo1-HX and ΔMCHC (rMCHC-MCHC) was 18.7 ± 13 vs. −50 ± 8.7 g/L (*p* < 0.001). A threshold of 8.6 fL for ΔMCV and −5.5 g/L for ΔMCHC could discriminate between Gardos and Piezo1-HX with 100% sensitivity and specificity, regardless of age, mutation or splenectomy status. Consequently, we showed that reticulocytes indices are useful to suggest Gardos-HX on blood count results, allowing to rapidly target these patients for gene analysis. In addition, these parameters may prove useful as a ‘functional tool’ in interpreting new *KCNN4* variants.

Gardos hereditary xerocytosis (Gardos-HX) is the most recently described hereditary hemolysis, also known as dehydrated stomatocytosis type II, or Gardos channelopathy (Glogowska et al., 2015; Rapetti-Mauss et al., 2015). Hereditary xerocytosis (HX) are rare dominant red cell membrane disorders initially characterized by K^+^ leak leading to decreased intracellular cationic content, loss of water and red cell dehydration (Delaunay, 2004). In most cases, HX is associated with gain of function heterozygous mutations in *PIEZO1* (Piezo1-HX). Piezo1 is a mechanosensitive cation channel that translates a mechanical force into a biological signal, mainly through a Ca^2+^ influx into red cells (Zarychanski et al., 2012; Albuisson et al., 2013; Andolfo et al., 2013). Gardos-HX is caused by heterozygous activating mutations in *KCNN4*, encoding the Ca^2+^-dependent K^+^ exporter Gardos channel. Functional studies have suggested that Gardos channel activation is the common effector of red cell dehydration, triggered either directly by *KCNN4* activating mutation or indirectly by Piezo1-dependant intracellular Ca^2+^ increase (Rapetti-Mauss et al., 2017; Caulier et al., 2018).

Gardos-HX diagnosis is difficult and often delayed because of the absence of typical clinical and biological phenotype. Indeed, it presents as a chronic hemolysis with negative red cell phenotypic investigations including osmolar gradient ektacytometry, which is normal or not specific. Diagnosis is made by genetic analysis, often performed after ruling out many other causes of hemolysis (King et al., 2015). It is important to distinguish Piezo1- and Gardos-HX because of several distinct clinical issues: non-spherocytic chronic hemolysis and risk of iron overload are common to both disorders, however, post-splenectomy thrombotic events and perinatal edemas without anemia are observed in Piezo1-HX, whereas anemia –including pre/neonatal anemia – are more severe in Gardos-HX (Fermo et al., 2017; Andolfo et al., 2018; Picard et al., 2019).

We report here that diagnosis of Gardos-channelopathy can be substantially sped up using reticulocyte indices. In a previous retrospective study including 12 Gardos and 91 Piezo1-HX cases, we have already described red cell parameters – Hb level, reticulocytes count, mean cell volume (MCV), mean cell hemoglobin concentration (MCHC) – in Gardos and Piezo1-HX (Picard et al., 2019). Specifically, we showed that MCV was not significantly different in both disorders, however, MCHC, which is in the normal range in subjects with Gardos channelopathy, was significantly higher in Piezo1-HX (Picard et al., 2019). These observations were also reported by others, they are consistent with data from osmotic gradient ektacytometry assays since these curves show a characteristic left-shifted dehydrated profile in Piezo1-HX but not in Gardos-HX indicating the absence of clear red cell dehydration features (Rapetti-Mauss et al., 2015; Picard et al., 2019). Here, we have extended these observations by analyzing reticulocyte indices in an enlarged series of 15 KCNN4-mutated cases (three new subjects, 12 already described) aged 1–59 years from eight families (two new families), that were compared to 79 Piezo1-HX subjects (49 families) with complete blood count records from the described cohort (Picard et al., 2019). Our primary objective was to better define how red cell and/or reticulocyte indices could be used in diagnosing HX. All patients gave their informed consent according to the Helsinki protocol, and this report followed the French regulations in terms of non-interventional retrospective study. HX diagnosis was based on clinical and biological data and a typical ektacytometry curve for the 79 Piezo1-HX patients, all of them carried at least one rare (MAF < 0.01) *PIEZO1* mutation, this mutation may be already described as associated with HX or not. In the 15 Gardos-HX patients, the ektacytometric profile was normal (*n* = 10) or atypical (*n* = 5) and therefore not contributive for diagnosis, genetic testing identified a *KCNN4* mutation that segregated with the disease in the eight families. Six Gardos-HX families (12 patients) were already described, one patient was found to carry a new KCNN4 mutation (c.965C > T, p.Ala322Val), in addition, three unreported subjects from two novel families were found to carry the recurrent p.Arg352His mutation. Overall, 11 cases from six families carried the recurrent p.Arg352His substitution and 4 cases from two families carried private KCNN4 mutations. The clinical, biological and genetic characteristics of KCNN4-mutated cases are summarized in Supplementary Table 1. All subjects had a complete blood count performed in the same lab on EDTA-blood samples within a 24 h/4°C delay after blood harvesting using an ADVIA2120 (Siemens^®^) analyzer, that provided specialized red cell and reticulocyte parameters. The ADVIA2120 analyzer measures MCV and MCHC using single cell analysis by dual angle laser scattering cytometry. On one channel, MCV and MCHC indices are measured on red cells, including reticulocytes as well as mature cells. On a second channel using a different set of reagents, reticulocyte counts and indices (rMCV, rMCHC) are distinguished from mature red cells (mMCV, mMCHC) by using oxazine 750 staining. All statistical analyses were performed using two-tailed *p* value and parametric tests. Statistical significance used was α = 0.05. For quantitative variables, we used Student’s *t*-test or one-way ANOVA test and Tukey *post hoc* analysis for multiparametric analysis. All numeric values were expressed as mean values ± SEM.

As already described, mean hemoglobin level was lower in Gardos vs. Piezo1-HX (103 ± 16 vs. 134 ± 19 g/L, respectively), MCV was not significantly different (98.8 ± 9 vs. 97.7 ± 8 fL), and MCHC was lower in Gardos compared to Piezo1-HX subjects (332 ± 13 vs. 354 ± 24 g/L, Student’s *t*-test, *p* < 0.01) (Figures 1A,B; Picard et al., 2019). Now, a focus on reticulocytes indices indicated that, quite surprisingly, Gardos-HX reticulocytes appeared smaller than expected compared with total red cells (95.1 ± 3 vs. 98.8 ± 9 fL) and their MCHC was higher (367 ± 16 vs. 332 ± 13 g/L). This was not the case in Piezo1-HX, where, as expected, reticulocytes were larger than red cells with a lower MCHC (113.7 ± 7 vs. 97.7 ± 8 fL and 329 ± 20 vs. 354 ± 24 g/L). Therefore, Gardos-HX reticulocytes appeared significantly smaller and had a significantly higher MCHC compared with Piezo1-HX (95.1 ± 3 vs. 113.7 ± 7 fL and 367 ± 16 vs. 329 ± 20 g/L, respectively) (Figures 1C,D). As a consequence, the differential MCV between reticulocytes and mature red cells ΔMCV (rMCV – mMCV) was −0.9 ± 5 in Gardos vs. 19.8 ± 3 fL in Piezo1-HX (*p* < 0.001), and the ΔMCHC (rMCHC – mMCHC) was 18.7 ± 3 in Gardos vs. −50 ± 8.7 g/L in Piezo1-HX (*p* < 0.001) (Figures 1E,F). We then used ROC curves to analyze the performance of these reticulocyte parameters in differentiating Gardos or Piezo1-mutated cases. The ΔMCHC and ΔMCV were found to be the best parameters (Supplementary Figure 1). Indeed, a value of 8.6 fL for ΔMCV and −5.5 g/L for ΔMCHC could discriminate both genotypes with a 100% sensitivity and 100% specificity with no overlap in this small series (Figures 1E,F). Because of the low number of Gardos-HX cases, these parameters should be further tested and validated in other labs before use in routine testing. Interestingly, these observations revealed correct for each individual subject, whatever the age, whether they carry the p.Arg352His recurrent mutation or a private mutation. We have not tested subjects carrying the p.Val282Met or p.Val282Glu substitutions, both associated with Gardos-HX, reticulocyte indices in these patients deserve to be investigated (Glogowska et al., 2015).

**FIGURE 1:**
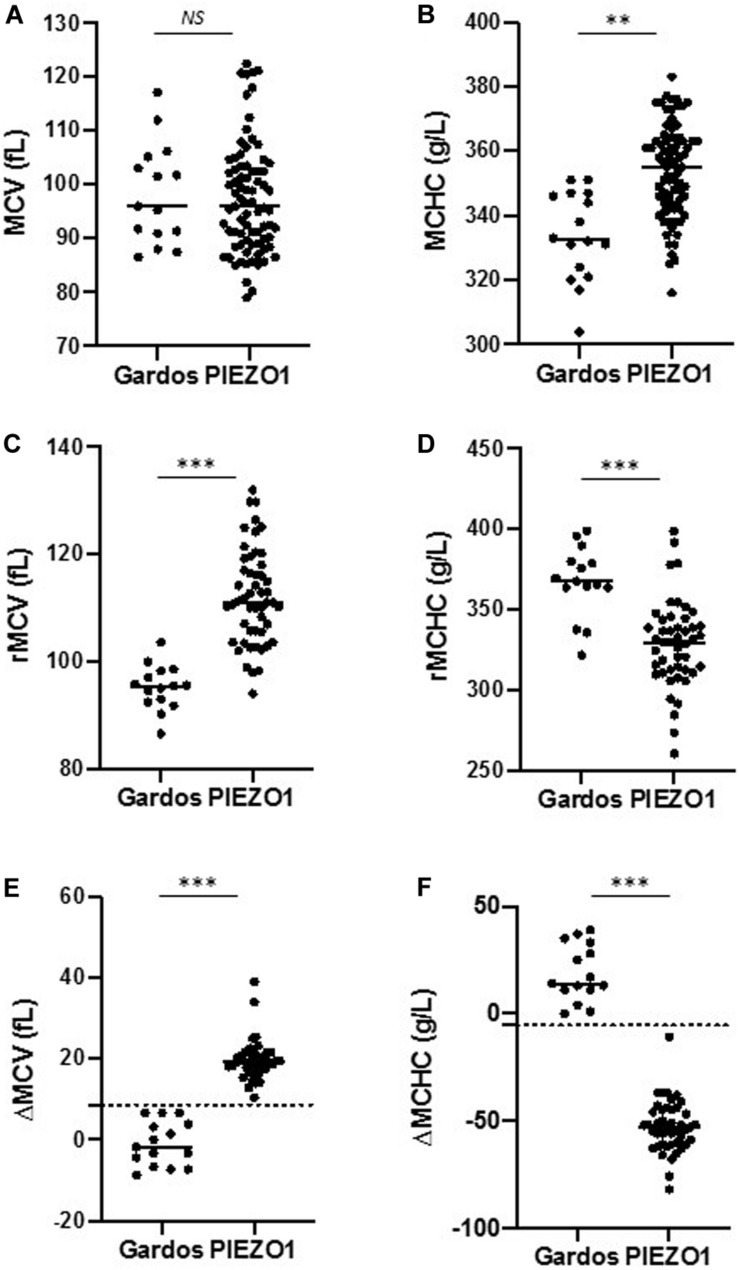
Red cell and reticulocytes indices in Piezo1- and Gardos-HX. **(A,B)** Comparison of MCV (fL) and MCHC (g/L) of all red blood cells (mature + reticulocytes) in Piezo1-HX (*n* = 79) and Gardos-HX (*n* = 15). **(C,D)** Comparison of MCV (fL) and MCHC (g/L) of reticulocytes in Piezo1-HX (*n* = 79) and Gardos-HX (*n* = 15). **(E,F)** ΔMCV (rMCV – mMCV) and ΔMCHC (rMCHC – mMCHC) parameters could discriminate between Gardos and Piezo1-HX, with cut-off values of 8.6 fL and –5.5 g/L, respectively (dashed line). *NS*, non-significant; ^∗∗^*p* < 0.01, ^∗∗∗^*p* < 0.001 as analyzed by Student *t* test. Normal range: MCV: 80–100 fL, MCHC: 310–360 g/L, rMCV: 92–120 fL, rMCHC: 270–330 g/L.

Then, since 7/15 Gardos vs. 8/79 Piezo1-HX cases were splenectomized, we asked whether splenectomy might influence red cell indices. As shown in Table 1, there was no significant difference in terms of rMCV and rMCHC between splenectomized and non-splenectomized subjects in both disorders. We observed that Gardos-HX splenectomized subjects had an increased MCV compared to non-splenectomized subjects (MCV: 107 ± 4 vs. 92 ± 3 fL, *p* < 0.001). Nonetheless, ΔMCV and ΔMCHC remained highly suggestive of Gardos-HX whether patients were splenectomized or not (Table 1 and Figure 2). In our setting, these new parameters revealed highly useful and reliable in order to target patients for *KCNN4* gene analysis as soon as the blood count was performed.

**TABLE 1:** Comparison of red cell, reticulocyte and differential indices in splenectomized vs. non-splenectomized Gardos and Piezo1-HX cases (mean ± SEM, *p*: student *T* test).

	**Normal range**	**Gardos-HX (*n* = 15)**			**Piezo1-HX (*n* = 79)**		
		**Splenectomy**			**Splenectomy**		
		**Yes**	**No**	***p***	**Yes**	**No**	***p***
*N*		7	8		8	71	
Hb (g/L)	120–160 (female) 130–180 (male)	918.7	1121.1	<0.05	123.717	124.812	0.24
Reticulocyte (G/L)	20–120	34115	17462	0.07	284135	307100	0.78
MCV	80–100	1074.2	922.7	<0.001	10311	967.1	0.25
rMCV (fL)	92–120	96.85.3	93.63.8	0.27	11410	1117	0.66
mMCHC (g/L)	310–360	34312	35323	0.5	36537	37021	0.84
rMCHC (g/L)	270–330	37213	36417	0.1	30529	33418	0.1
ΔMCV (fL)	–	−5.81.9	3.52.5	<0.001	16.36	19.66	0.05
ΔMCHC (g/L)	–	289	106	<0.001	−65.414	−52.48.6	0.21

*In Gardos-HX, ΔMCV was significantly lower and MCV and ΔMCHC were significantly higher in splenectomized subjects, in splenectomized or non-splenectomized cases, the differential indices remained discriminant for Gardos or Piezo1-HX.*

**FIGURE 2:**
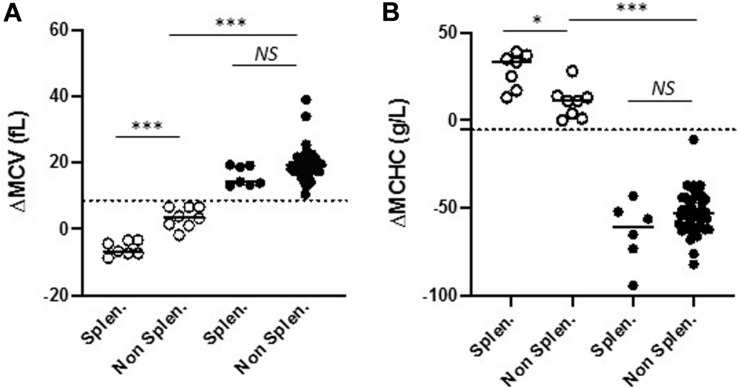
Comparison of ΔMCV **(A)** and ΔMCHC **(B)** in splenectomized and non-splenectomized patients in Gardos-HX (o) and Piezo1-HX (•). The cut-off value of 8.6 fL and –5.5 g/L, respectively are indicated (dashed line). These differential indices could discriminate between Gardos and Piezo1-HX whether subjects were splenectomized or not. *NS*, non-significant; ^∗^*p* < 0.05, ^∗∗∗^*p* < 0.001 as measured by one-way ANOVA test and Tukey *post hoc* analysis for multiparametric analysis.

Reticulocyte indices are not widely used in diagnosis, although reticulocytes hemoglobin content measured by most hematology analyzers has proved to be helpful in diagnosis of iron deficient anemia (Brugnara et al., 2006; David et al., 2006). Reticulocytes are immature cells, their volume is larger compared with mature red cells and their hemoglobin concentration lower, consistent with rMCV and rMCHC reference range provided by the manufacturer (rMCV: 92.0–120.0 fL, rMCHC: 270–330 g/L in adults) (Thomas, 2008). Currently, most blood cell analyzers do not measure rMCV and rMCHC. Whether red cell indices obtained on other systems using different optic or impedance-based technologies might allow to target Gardos-HX should be evaluated. The ADVIA^®^ technology measures individual red cell volume and hemoglobin content by cell light scattering after isovolumetric spherization. In this setting, Gardos-HX reticulocytes appeared small and dehydrated, they react differently from reticulocytes in Piezo1-HX or in other hemolytic anemia, acquired or hereditary, whether they are related to hemoglobin, enzyme or other membrane defects. This specific behavior of Gardos-HX reticulocytes might be due to an ionic imbalance induced by the *KCNN4* mutation *per se* or due to other differences in the membrane structure or composition. A difference in Gardos channel concentration at the membrane in reticulocytes compared with mature red cells might also account for this observation, to our knowledge, this point has not been studied before. Alternately, the interaction of the Gardos channel with proteins present at a different level in reticulocytes may be involved. Finally, we cannot rule out a possible *in vitro* artifact due to the ADVIA^®^ technology. Although we cannot infer that this *in vitro* observation does reflect reticulocytes properties *in vivo*, we hypothesize that dehydration predominates in immature cells, i.e., reticulocytes in Gardos-HX while mild or absent in mature red cells. Because reticulocytes represent a minor fraction of circulating red cells, this would account for the normal MCHC reported by several teams in Gardos-HX as well as the non-dehydrated ektacytometry profile. In Piezo1-HX, we propose that dehydration predominates in mature red cells and accounts for the observed high MCHC and dehydrated ektacytometric profile.

In conclusion, these data revealed a new feature distinguishing Gardos and Piezo1-HX. That a simple analysis of red cell and reticulocyte indices on a blood count were able to target each individual Gardos-HX subject was quite unexpected. Although the cause of this specific behavior of Gardos-HX remains to be established, it provides a very useful and cost-free tool for labs using ADVIA analyzers in order to suggest Gardos-HX and speed up diagnosis by *KCNN4* gene analysis. In addition, ΔMCV and ΔMCHC may prove useful as a “functional tool” in interpreting new *KCNN4* variants when Gardos-HX is suspected.

## Data Availability Statement

The original contributions presented in the study are included in the article/Supplementary Material, further inquiries can be directed to the corresponding author.

## Ethics Statement

The studies involving human participants were reviewed and approved by the French National Commission on Informatics and Liberty (CNIL). Written informed consent to participate in this study was provided by the participants’ legal guardian/next of kin.

## Author Contributions

VPi and LG designed the study and wrote the manuscript. LB and LM-H performed genetic studies. VPi, MD, VPr, BJ, and JD analyzed the data. LG, CG, and FG followed patients. All authors contributed to the article and approved the submitted version.

## Conflict of Interest

The authors declare that the research was conducted in the absence of any commercial or financial relationships that could be construed as a potential conflict of interest.
