# Supplementary material for: Rapid Gardos Hereditary Xerocytosis Diagnosis in 8 Families Using Reticulocyte Indices
Source: Front Physiol. 2021 Jan 14;11:602109. doi: 10.3389/fphys.2020.602109 (PMC7841495; doi:10.3389/fphys.2020.602109)
Supplement: Supplementary file 1 [file Table_1.pdf]

Supplemental Table 1: Characteristics of 15 Gardos-HX cases at diagnosis. (Y yes, n no, OGE: osmolar gradient ektacytometry , N Normal, A atypical). * undescribed cases

**Family** - member	Normal range	**1**-1	**1**-2	**1**-3	**2**-1	**3**-1	**3**-2	**4**-1	**4**-2	**5**-1	**6**-1	**6**-2	**6**-3	**7**-1*	**7**-2*	**8**-1*
**Mutation**		c.1109_1119+17del			p.Ala322Val	p.Arg352His										
**Age**		59	27	25	37	26	1	47	4	16	2	36	33	24	58	33
**Sex**		M	F	M	M	F	M	M	F	M	M	F	F	M	M	M
**Splenomegaly**		-	-	Y	-	Y	n	Y	n	n	Y	-	-	-	-	Y
**Splenectomy**		Y	Y	n	Y	n	n	n	n	n	n	Y	Y	Y	Y	n
**Cholelithiasis**		Y	Y	Y	Y	Y	n	Y	n	n	n	Y	Y	nd	nd	n
**Iron overload**		Y	Y	nd	Y	n	n	Y	Y	n	n	Y	Y	Y	Y	Y
**Iron chelation**		Y	n	nd	Y	n	n	Y	n	n	n	Y	Y	Y	Y	n
**Transfusions**		n	n	n	Y	n	n	n	Y	n	Y	Y	Y	n	n	n
**Hb level (g/L)**	120-160 (female)<br>130-180 (male)	82	91	108	86	124	94	120	95	122	97	82	94	97	118	133
**MCV (fL)**	80-100	103	102	91	106	96	88	95	87.5	91	87	112	105	117	105	102
**MCHC (g/L)**	310-360	324	304	321	331	347	351	333	344	320	347	332	317	338	346	331
**Reticulocytes (G/L)**	20-120	192.5	162	118	500	96	250	119	208	75	255	221	248	346	721	254
**rMCV (fL)**	92-120	92.1	94.5	95.4	95.8	92.9	86.5	97.4	88.3	98.5	90.2	107.4	92.4	103.6	91.8	99.9
**rMCHC (g/L)**	270-330	380	336	338	364	396	370	364	390	322	366	368	376	379	399	365
**Δ MCV (fL)**	–	-3.4	-4.4	6.7	-6.7	1.5	-1.8	3.2	1	6.7	6.6	-3.3	-8.7	-7.3	-7.3	3.9
**Δ MCHC (g/L)**	–	36	33	14	17	13	29	11	11	0	4	14	25	39	37	0,1
**OGE profile**		N	N	N	A	N	A	N	A	N	N	N	N	A	A	N
