# Supplementary figures and images for: Rapid Gardos Hereditary Xerocytosis Diagnosis in 8 Families Using Reticulocyte Indices
Source: Front Physiol. 2021 Jan 14;11:602109. doi: 10.3389/fphys.2020.602109 (PMC7841495; doi:10.3389/fphys.2020.602109)

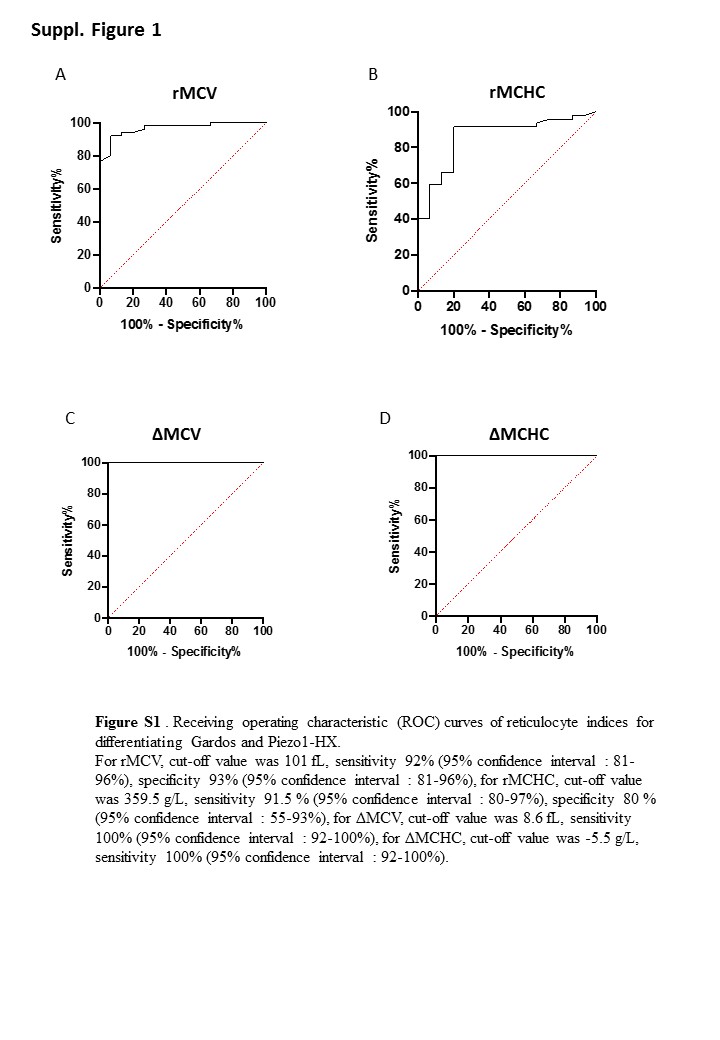

Supplement: Supplementary file 2 [file Image_1.JPEG]
